# Implementation of an Educational iPad Application for Patients With Chronic Hepatitis B

**DOI:** 10.3389/fpubh.2019.00372

**Published:** 2019-12-10

**Authors:** Phil Ha, Rattanak Hean, Patrick Tang, Audrey Choy, Udit Thakur, Anouk Dev

**Affiliations:** ^1^Department of Gastroenterology and Hepatology, Monash Health, Melbourne, VIC, Australia; ^2^Faculty of Medicine, Nursing and Health Sciences, Monash University, Melbourne, VIC, Australia

**Keywords:** hepatitis, iPad, education, health knowledge, public health

## Abstract

Chronic Hepatitis B (CHB) contributes to a high public health burden in Australia from chronic liver disease and hepatocellular carcinoma. Health literacy impacts on multiple aspects of long term management, including surveillance and long term follow up. We designed and implemented a multilingual educational iPad application for outpatients to use while in the clinic waiting room. The application employed an interactive and multimodal approach to education. It utilized graphics, audio and text to convey practical information regarding transmission of disease, long term complications, treatment and surveillance. Participants were recruited from a tertiary liver clinic and assigned to either standard treatment (routine clinical consult only) or the iPad group (clinical consult and additional education with the iPad app). There were 54 participants (control *n* = 29, iPad *n* = 25). Knowledge was assessed at baseline, secondly after the clinician appointment and finally at 6 months. Median follow up time was 6.1 months (range 0–18 months) and 87% of participants completed the final survey. At baseline, there was no difference in age, gender, proportion of newly referred patients, or use of antivirals. Baseline knowledge was similar in the two groups (61.4 vs. 55.1%, *p* = 0.33). The iPad group scored significantly higher after the first consult (79.5 vs. 61.5%, *p* = 0.0005). This improvement remained significant by the end of follow up (72.6 vs. 61.0%, *p* = 0.0472). To conclude, interactive education with iPads may be an effective way to improve patient knowledge.

## Introduction

Chronic Hepatitis B (CHB) contributes to an increasing public health burden in Australia due to its complications of chronic liver disease (CLD) and hepatocellular carcinoma (HCC). In Australia, it is estimated that there are 230,000 people with CHB, with a disproportionately high prevalence in patients from Indigenous, Asian-Pacific and African backgrounds as well as those who inject drugs or men who have sex with men (1). In our catchment area in eastern Melbourne, there is a large presence of CHB patients from Vietnam, China, Cambodia, Sudan and Afghanistan.

Poor health literacy has been observed in patients with CHB in a number of different studies (2–4). This likely contributes to the high rates of non-adherence to antiviral medications and the suboptimal outpatient clinic retention rates that have been observed in other studies (5–7). Patient barriers that may contribute to these lower health literacy and poor health outcomes are low levels of English proficiency, stigma and perceptions of CHB, attitudes and level of education. Anecdotally in our tertiary clinic, a significant barrier to further health education from healthcare providers has been time constraints and high patient loads, with follow up consultations focusing mainly on antiviral therapies. We find that patient education of CHB relies heavily on patients' primary care physician. New methods of delivering health education are required to further improve patient understanding of CHB by overcoming these barriers.

From previous research we have undertaken at our liver clinic, many patients with CHB had indicated that they wanted further information on CHB via either the internet, pamphlets or in video format (6).

Tablet based education modules have been deployed in several clinical settings in recent years with variable results. Short term studies in Dermatology, Endocrinology and Oncology have been performed suggesting that this medium of education is well-received and feasible, however long term outcomes were not assessed (8–10). Interestingly, age was not a significant barrier to iPad application adoption, and there is data to suggest that clinical outcomes following tablet based education are non-inferior in the medium term (9, 11, 12).

## Method

The aim of this pilot study was to design, develop and trial a novel approach using a multilingual and audio-visual iPad application in the outpatient waiting room to supplement patient education. We aimed to assess its effectiveness as an adjunct in improving patient knowledge over a short and medium follow up period.

**Figure 1:**
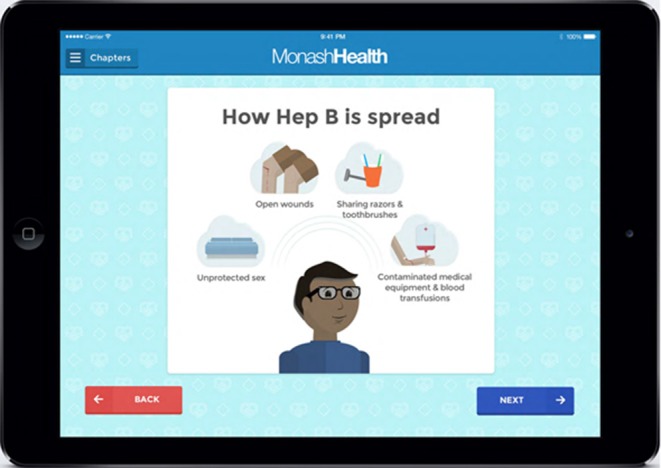
Screenshot of a chapter on Hepatitis B Transmission in our educational iPad application.

We designed an educational iPad application in five languages (English, Khmer, Dari, Vietnamese and Mandarin) that educated patients about different aspects of CHB. Health knowledge regarding the transmission, prevention, complications, surveillance, and management of CHB were addressed sequentially in the application. Patients were able to revisit previous sequences as often as required. Voice-over narration and captions were available in the chosen language, as well as cartoon animations to maximize knowledge retention (Figure 1). The iPad application was available for use for 20 min prior to attending their scheduled clinic review.

Participants were identified from a tertiary liver clinic and invited to participate in this study. Inclusion criteria were patients who had CHB and fluency in at least one of the five languages available in the application. We identified two groups of patients—those who had been newly referred to the clinic by their GP for management of CHB, and those who were already seeing the liver clinic for CHB. Written consent to participate in this study was obtained with the aid of interpreters as required.

Participants were then randomized to either a control or an intervention group. Throughout the follow up period, participants knowledge of CHB was assessed three times (baseline, post-consultation, and follow up) using a questionnaire. All patients underwent a baseline questionnaire assessing knowledge of CHB prior to their first clinical contact. Following this questionnaire, the iPad group were given iPads to review the educational CHB material while control patients continued to wait in the waiting room. Both groups then proceeded to see their respective hepatologist, who were not informed of which patients had used the iPad app. Following this consultation, participants completed the post-consultation questionnaire prior to leaving the clinic. The long-term questionnaire was completed at the conclusion of the follow up period (~6 months) either in person, on the telephone or online.

Results were analyzed using STATA (13). Baseline characteristics between control and intervention groups were compared using *t*-test and chi-square tests. There were 19 questions assessing CHB knowledge in total: vaccination (*n* = 2), vertical transmission (*n* = 3), horizontal transmission (*n* = 8), liver related complications (*n* = 2), social stigma (*n* = 1) and need for follow up (*n* = 3) (Appendix 1 in Supplementary Material). Individual questions from the survey were weighted to a score of 1–3 based on their clinical significance, with the total score being presented as a percentage out of 40. We assigned greater weight to knowledge that would have a more impact on patient management and quality of life.

Questions that assessed the importance of long term follow up and complications of CHB were assigned 3 points, as we believed this to be a large barrier to long term follow-up. Questions that assessed the horizontal transmission risks were graded between 1 and 3 points based on the daily exposure to this risk and the effect on quality of life. For example, we scored the question assessing knowledge that sharing food and drink to be safe as 3 points, and the risk of sharing earrings as 1 point.

This study was approved by the local governing ethics committee.

## Results

There was a total of 54 participants included this study (control *n* = 29, iPad *n* = 25). At baseline, there was no difference in age, gender, newly referred patients, use of antivirals or proportion who chose English as their first preference (Table 1).

**Table 1:** Baseline characteristics.

	**Control (*n* = 29)**	**Ipad (*n* = 25)**	
Age (years)	46.5 ± 11.2	44.0 ± 12.5	*p* > 0.05
Male (%)	48.3	56.0	*p* > 0.05
New referrals (%)	44.8	48.0	*p* > 0.05
On Antiviral therapy (%)	17.2	32.0	*p* > 0.05
Diagnosis of HBV > 5 years prior (%)	75.9	84.0	*p* > 0.05
English as 1st preference (%)	58.6	56.0	*p* > 0.05

Ninety-three percentage of patients (*n* = 50) completed the post-consultation questionnaire, and 87% (*n* = 47) completed the long term questionnaire. Median follow up time was 6.1 months (range 0–18), and patients attended a median of 2 clinic appointments during this time.

### Baseline Results

At baseline, there was no difference in knowledge between the iPad and control groups with overall scores (61.4 vs. 55.2% *p* = 0.32). Patients who used the English version the questionnaire or the English iPad application (*n* = 31) scored higher than those who used non-English versions (Khmer *n* = 6, Mandarin *n* = 11, Vietnamese *n* = 6) (63.7 vs. 50.3%, *p* = 0.04). There was no difference in overall knowledge in patients newly referred to the clinic compared to existing patients (56.0 vs. 60.5%, *p* = 0.47).

Participants scored highest in identifying the complications of CHB, and lowest in questions regarding vaccinations (Table 2). Forty-three percentage of participants were unsure about or believed that sharing food and drink could spread the hepatitis b virus, and 35% thought the same of coughing/sneezing. Sixty-five percentage and 67% of all participants were not aware of the risk of transmission when sharing nail clippers and earrings, respectively. Eighty-five percentage of patients believed that CHB requires long term surveillance and follow up, and 55% of patients understood that having a diagnosis of CHB would not be grounds for losing their employment.

**Table 2:** Mean weighted scores achieved by participants throughout the study.

	**Baseline**		**Post 1st consult**		**Long term follow up**	
	**Control** ***n* = 29 (%)**	**iPad** ***n* = 25 (%)**	**Control** ***n* = 27 (%)**	**iPad** ***n* = 23 (%)**	**Control** ***n* = 25 (%)**	**iPad** ***n* = 22 (%)**
Vaccination	44.8	50.1	49.4	58.7	57.3	50.0
Horizontal transmission	51.8	64.0	57.9	82.6	63.3	75.3
Vertical transmission	47.2	54.0	58.0	72.5	51.3	58.3
Complications	75.9	74.0	83.3	91.3	66.0	88.6
Management	57.3	56.0	59.7	76.6	60.0	75.0
Stigma	48.3	64.0	48.0	79.6	72.0	68.2
Total	55.2	61.4	61.5	79.5	61.0	72.6

### Post-consultation Results

**Figure 2:**
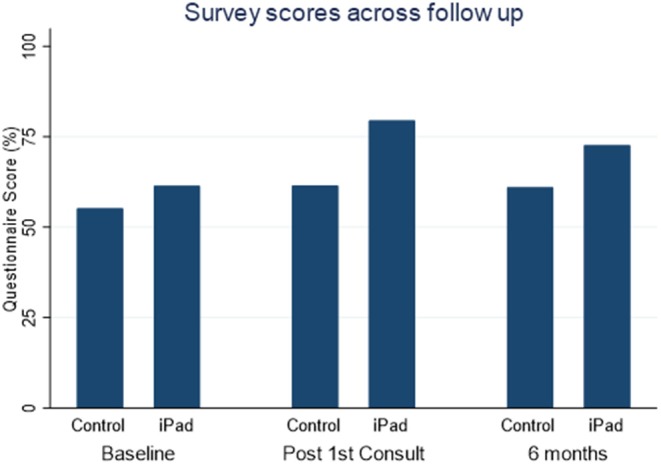
Comparison of total scores on knowledge questionnaires throughout follow up.

The iPad group demonstrated higher overall knowledge compared with the control group in the post first consultation questionnaire (79.5 vs. 61.5%, *p* < 0.0005; Figure 2).

Patients who used the non-English version of the iPad application had more substantial improvements in knowledge following the clinician consult compared with both those who used the English version (absolute increase of +27.2% vs. +11.3%, *p* = 0.045) and those non-English speaking in the control group (+27.2% vs. 5.9%, *p* = 0.007).

### Long Term Outcomes

At the end of follow up, the iPad group scored higher than the control group in overall CHB knowledge (72.6 vs. 61.0%, *p* = 0.047; Figure 2), and this was significant in areas concerning complications associated with CHB (88.6 vs. 66.0%, *p* = 0.026) and management (75.0 vs. 60.0%, *p* = 0.04).

Non-English speaking patients who used the iPad application showed no difference in knowledge compared with non-English speaking patients in control group (63.2 vs. 61.7%).

Newly referred patients who used the iPads had improvements in their overall knowledge by the end of follow up compared to their own baseline (80.3 vs. 64.8%, *p* = 0.020). Newly referred patients in the control group trended toward a significant improvement in knowledge scores by the completion of the study compared to their baseline (80.3 vs. 68%, *p* = 0.079).

There was no difference in test scores at the end of follow up in those who complete the questionnaire on or before 6 months, and those after 6 months.

## Discussion And Conclusion

### Discussion

#### Poor Baseline Health Knowledge

Poor health literacy remains a barrier to the long-term management of patients with CHB. This has been shown in multiple populations, and our baseline questionnaire demonstrates similar findings.

At baseline, only 56% of patients were aware that a vaccine for CHB existed. Of these patients, 57% were aware that this vaccination is subsidized by the Australian Government. Interestingly, there was high expectation of needing antiviral therapy at baseline. Only 34% of patients who were not on antiviral therapy correctly believed that they did not need antiviral therapy; the majority were not sure or believed that medications would be beneficial.

We were not surprised to find that patients who completed non-English versions of the questionnaire scored lower than their counterparts who chose English as their first preference. Language barriers, level of education and different cultural attitudes toward chronic disease likely contributed to this difference. This is consistent with the experience of other Australian centers (2–4).

There was no significant difference in knowledge between those who were newly referred to the clinic and those who were already managed by a hepatologist at the clinic, which suggests that further interventions such as additional educational resources may be valuable in improving health literacy in patients with CHB.

#### Development of the Application

Initially, the application was designed in English and we aimed to address topics that would be clinically relevant to patients. We focussed on the explanation of the prevention and transmission of HBV, indications for antiviral therapy, long term surveillance and potential complications of the virus. Once the English narration and subtitles were completed, we translated the app into four different languages—Khmer, Dari, Vietnamese, and Mandarin. Epidemiological information specific to Australia and to each country was included in the application (for example, if a patient chose the Vietnamese language option, epidemiological data from Vietnam and Australia was displayed). We believe that presenting health information in a multilingual audio, visual and text format is ideal to optimize knowledge retention, especially in a population of patients who have lower literacy rates.

Another goal of the app was to introduce patients to the multidisciplinary nature of CHB management, thus also reinforcing the importance of regular appointment with phlebotomy and sonography services.

#### Issues With Implementation

Using the iPad application in our clinics was well-received by patients. In our study, we used a separate room adjacent to the waiting room to allow patients to complete the questionnaire and use the iPads alone. Implementation of similar apps in open waiting rooms will need address this issue. If side-rooms are not readily available, we believe that using headphones and physical tablet privacy screen filters may be a reasonable option.

Although we did not formally assess the participants' technical or digital skill, we found that the iPads were able to be used without difficulty by most participants.

#### Improvements in Knowledge

Our data suggests that using the iPad app resulted in significant improvements in knowledge regarding CHB. We designed the questionnaire to test for practical knowledge regarding the management of CHB from a patient perspective, and therefore improvements in scores are encouraging. Knowledge regarding how the virus is and is not transmitted could have a large impact on some patient's families and lifestyles, while from a management perspective, better understanding of the complications of CHB could perhaps improve clinic and medication retention rates.

There was a marked improvement in knowledge immediately after using the iPad application, however this improvement reduced over time. This decay is not unexpected, and perhaps regular education is required for optimal levels of knowledge retention.

Patients who preferred non-English versions of the application benefitted most in terms of knowledge gained as demonstrated by significant greater improvements in post-questionnaire scores, therefore highlighting the effectiveness of the education app in patients more likely to have lower literacy. However, knowledge scores were similar between the control and iPad non-English speaking groups at the end of follow up. We also see that patients who were newly referred to the clinic demonstrated sustained improvements in knowledge across the follow up period. Again, this is not a surprising finding given that the newly referred patients will perhaps be more eager to learn about their disease, are younger than those who are already known to the clinic (41.5 years vs. 48.7, *p* = 0.029).

#### Future Direction

Smart devices are now widely accepted and used by people of all ages. From a health education perspective, these offer great opportunities in improving patient knowledge and allowing for more holistic care. We believe that applications can be used in the waiting room as an adjunct to traditional means of health education. Furthermore, there is great potential for using these applications to directly influence specialist consultations. Clinician access to questionnaire data would allow immediate identification of knowledge deficiencies and patient misconceptions about their disease, thus enabling a more targeted consult and personalized approach to patient care. Future studies will explore the repeated administration of apps to facilitate sustained learning, while also exploring the long-term adherence and outcomes for patients with CHB.

#### Practical Implications

The use of iPad assisted patient education in the waiting room presents great potential. It can be implemented easily in a busy tertiary clinic with minimal interruption to patient flow. This could be a cost effective way of improving patient knowledge, and potentially reducing long term issues in this chronic disease.

#### Limitations

The findings of our study are limited for a number of reasons. Firstly, we followed a small study sample over the study period. Subgroup analyses performed within these samples are of small numbers, and the data must be interpreted cautiously. There was a variable follow up time, often due to difficulty contacting patients. This did, however, mean that we acquired even longer term results for some patients. We did not assess the reading literacy of participants in this study. This may impact on outcomes, however we aimed to minimize the reliance on reading literacy by implementing audio and visual educational content.

Although our study demonstrated modest improvements in patient knowledge of hepatitis B, it was not powered to directly assess changes in transmission rates, adherence to medications and follow up. Larger studies are required to directly determine the efficacy of this application in improving these important health outcomes in patients with CHB.

## Conclusion

We have developed and successfully implemented a supplementary iPad based health education application for patients with chronic Hepatitis B attending a tertiary liver clinic. A single episode of the iPad application use was associated with sustained improvement in disease specific knowledge over our study period.

## Data Availability Statement

The datasets generated for this study are available on request to the corresponding author.

## Ethics Statement

The studies involving human participants were reviewed and approved by Monash Health Human Research Ethics Committee. The patients/participants provided their written informed consent to participate in this study.

## Author Contributions

PH, RH, PT, AC, and UT were involved in the planning of this project and collection of data. PH analyzed this data and wrote the manuscript. AD was involved in planning this project and supervised its progress.

### Conflict of Interest

The authors declare that the research was conducted in the absence of any commercial or financial relationships that could be construed as a potential conflict of interest.
