# Supplementary material for: Implementation of an Educational iPad Application for Patients With Chronic Hepatitis B
Source: Front Public Health. 2019 Dec 10;7:372. doi: 10.3389/fpubh.2019.00372 (PMC6916629; doi:10.3389/fpubh.2019.00372)
Supplement: Supplementary file 1 [file Data_Sheet_1.docx]

**Appendix 1: Questionnaire used to assess chronic hepatitis B disease related knowledge**

|  | Points assigned for correct response |
| --- | --- |
| **Vertical Transmission**   - Possible - Reducible - Breastfeeding | 3  1  2 |
| **Vaccination**   - Is there a vaccination for hepatitis b? - Is the vaccination free? | 2  1 |
| **Horizontal Transmission**   - Sharing razor blades - Sharing food and drink - Hugging and Kissing - Sexual intercourse - Coughing and sneezing - Sharing nail clippers - Touching exposed blood - Sharing earrings | 1  3  3  2  3  1  2  1 |
| **Liver related complications**   - Liver failure - Liver cancer | 3  3 |
| **Social Stigma**   - Can you lose your job for having hepatitis b? | 1 |
| **Management issues**   - Do you need medicine to treat the hepatitis b? - Will this medicine cure the hepatitis b completely? - Do you still need follow up with the doctor if the hepatitis b is inactive? | 3  2  3 |
|  | Total Score: 40 |
